# Regulation of Ketogenic Enzyme HMGCS2 by Wnt/β-catenin/PPARγ Pathway in Intestinal Cells

**DOI:** 10.3390/cells8091106

**Published:** 2019-09-19

**Authors:** Ji Tae Kim, Chang Li, Heidi L. Weiss, Yuning Zhou, Chunming Liu, Qingding Wang, B. Mark Evers

**Affiliations:** 1 Markey Cancer Center, University of Kentucky, Lexington, KY 40536 USA; ji.tae.kim@uky.edu (J.T.K.); Heidi.Weiss@uky.edu (H.L.W.); yuning.zhou@uky.edu (Y.Z.); Chunming.Liu@uky.edu (C.L.); 2 Department of Surgery, University of Kentucky, Lexington, KY 40536 USA; Chang.Li@uky.edu; 3 Department of Molecular and Cellular Biochemistry, College of Medicine, University of Kentucky, Lexington, KY 40536-0509, USA

**Keywords:** ketogenesis, HMGCS2, Wnt/β-catenin pathway, PPARγ, intestinal cells, β-hydroxybutyrate

## Abstract

The Wnt/β-catenin pathway plays a crucial role in development and renewal of the intestinal epithelium. Mitochondrial 3-hydroxy-3-methylglutaryl-CoA synthase 2 (HMGCS2), a rate-limiting ketogenic enzyme in the synthesis of ketone body β-hydroxybutyrate (βHB), contributes to the regulation of intestinal cell differentiation. Here, we have shown that HMGCS2 is a novel target of Wnt/β-catenin/PPARγ signaling in intestinal epithelial cancer cell lines and normal intestinal organoids. Inhibition of the Wnt/β-catenin pathway resulted in increased protein and mRNA expression of HMGCS2 and βHB production in human colon cancer cell lines LS174T and Caco2. In addition, Wnt inhibition increased expression of PPARγ and its target genes, *FABP2* and *PLIN2*, in these cells. Conversely, activation of Wnt/β-catenin signaling decreased protein and mRNA levels of HMGCS2, βHB production, and expression of PPARγ and its target genes in LS174T and Caco2 cells and mouse intestinal organoids. Moreover, inhibition of PPARγ reduced HMGCS2 expression and βHB production, while activation of PPARγ increased HMGCS2 expression and βHB synthesis. Furthermore, PPARγ bound the promoter of HMGCS2 and this binding was enhanced by β-catenin knockdown. Finally, we showed that HMGCS2 inhibited, while Wnt/β-catenin stimulated, glycolysis, which contributed to regulation of intestinal cell differentiation. Our results identified HMGCS2 as a downstream target of Wnt/β-catenin/PPARγ signaling in intestinal epithelial cells. Moreover, our findings suggest that Wnt/β-catenin/PPARγ signaling regulates intestinal cell differentiation, at least in part, through regulation of ketogenesis.

## 1. Introduction

Mammalian intestinal epithelium is maintained by a continuous renewal process comprised of proliferation, migration, differentiation, and apoptosis [1,2]. A variety of signaling molecules or pathways, including Wnt/β-catenin signaling, are involved in controlling this process [3,4]. A breakdown of this highly integrated and homeostatic process in the intestinal epithelium is related to several pathological disorders including colorectal cancer (CRC) and inflammatory bowel disease [5,6,7].

The Wnt/β-catenin pathway plays an essential role in early development and tissue maintenance of adults through regulating T-cell factor/lymphoid enhancer-binding, factor-dependent transcription of its target genes [8,9]. Wnt/β-catenin signaling is strongly associated with the aforementioned homeostatic processes through stem cell maintenance, differentiation inhibition, migration regulation, and localization of epithelial cells along the crypt–villus axis in the intestinal epithelium [4,10,11]. In particular, alterations of Wnt/β-catenin signaling by genetic mutations or epigenetic silencing have been implicated in neoplastic transformation and cancer progression within the intestine [11,12,13].

Peroxisome proliferator-activated receptors (PPARs), which function as transcription factors, are a group of ligand-activated nuclear receptors consisting of three subtypes of PPARs (α, β/δ, and γ) [14,15]. PPARs play a broad range of biological roles in cell proliferation, differentiation, and apoptosis, as well as metabolism. PPARα, known as a master regulator of lipid metabolism, controls ketogenesis via transcriptionally regulating the expression of mitochondrial 3-hydroxy-3-methylglutaryl-CoA synthase 2 (HMGCS2) [16,17]. PPARγ, which is also a key regulator of lipid and glucose metabolism, counteracts the effect of Wnt/β-catenin signaling in multiple human diseases including CRC, and high-fat-diet-induced PPARγ induces HMGCS2 expression in cardiomyocytes [14,18,19,20,21].

HMGCS2 is the rate-limiting enzyme for ketogenesis, which leads to the production of ketone bodies including β-hydroxybutyrate (βHB) [22]. Expression and activity of HMGCS2 are regulated by several transcription factors, such as PPARs, and by posttranslational modifications including acetylation [17,23]. In particular, it has been reported that HMGCS2 expression was repressed by c-Myc, which is activated by the Wnt/β-catenin pathway, in colon cancer cells [24]. In human cancers, the expression level and cellular function of HMGCS2 are controversial depending on tissue types; increased expression of HMGCS2 has been observed in breast and prostate cancers, whereas diminished expression has been shown in esophageal squamous cell carcinoma, hepatocellular carcinoma, and CRC [24,25,26,27,28]. Recently, our laboratory demonstrated that HMGCS2 contributes to intestinal cell differentiation [29]. Moreover, it was shown that strong staining of HMGCS2 was detected in the villus, the most differentiated intestinal region [29]. This expression pattern of HMGCS2 is inversely associated with the activation level of Wnt/β-catenin signaling, which is high in the bottom part of the crypt and decreased along the crypt–villus axis [30].

In this study, we showed that Wnt/β-catenin/PPARγ signaling regulates HMGCS2 expression and βHB production in intestinal cells. Moreover, we found the inhibitory role of the HMGCS2 in glycolysis, which regulates intestinal cell differentiation. Dysregulated Wnt/β-catenin signaling may result in aberrant regulation of ketogenesis and an imbalance in proliferation and differentiation patterns within the intestinal crypts, which is associated with a number of intestinal pathologies.

## 2. Materials and Methods

### 2.1. Cell Culture, Treatment, and Transfection

The human colon cancer cell lines used in these studies, LS174T and Caco2, were obtained from the American Type Culture Collection (ATCC, Manassas, VA, USA). LS174T and Caco2 cells were cultured in EMEM supplemented with 10% FBS and in MEM with 15% FBS, 1% sodium pyruvate, and 1% nonessential amino acids. The cells were maintained at 37 °C in a humidified 5% CO_2_ incubator. Authentication and mycoplasma contamination tests were performed as described previously [29]. Recombinant Wnt3a, an activating ligand of the Wnt/β-catenin pathway and iCRT3, an inhibitor of β-catenin responsive transcription [31], were obtained from PeproTech (Rocky Hill, NJ, USA) and EMD Chemicals (San Diego, CA, USA), respectively. Rosiglitazone (RGZ; a PPARγ agonist) and T 0070907 (T007; a PPARγ antagonist) were purchased from Tocris Bioscience (Bristol, UK). 2-Deoxy-d-glucose (2-DG), a glucose analog that inhibits glycolysis, was obtained from Sigma-Aldrich (St. Louis, MO, USA). Transfection with ON-TARGETplus SMARTpool siRNAs for NTC (catalog #, D-001810-10), β-catenin (catalog #, L-003482-00), c-Myc (catalog #, L-003282-02), PPARγ (catalog #, L-003436-00), and HMGCS2 (catalog #, L-010179-00) (Dharmacon, Lafayette, CO, USA) was performed using Lipofectamine RNAiMAX (Invitrogen, Carlsbad, CA) according to the manufacturer’s protocol. Adenoviruses encoding GFP (Ad-GFP, control) and human c-Myc (Ad-c-Myc) were purchased from Vector BioLabs (Malvern, PA, USA).

### 2.2. Western Blot Analysis

Western blotting was carried out as described previously [32]. The antibodies for HMGCS2 and PPARγ were obtained from Abcam (Cambridge, MA, USA). The antibodies for β-catenin and β-actin were purchased from Sigma-Aldrich. The antibodies for Axin2 and c-Myc were acquired from Cell Signaling (Danvers, MA, USA). The anti-PPARα antibody was purchased from Santa Cruz Biotechnology (Santa Cruz, CA, USA). Densitometric quantification from three separate experiments was done using ImageJ.

### 2.3. Intestinal Organoid Culture

Isolation and culture of mouse small intestinal organoids was carried out as described previously [33]. Briefly, primary crypts were collected from the small intestinal mucosa in mice. Isolated crypts were mixed with Matrigel (BD Biosciences, San Jose, CA, USA) and cultured in basic culture medium (ENR) containing advanced DMEM/F12 medium with 50 ng/mL EGF (PeproTech), 100 ng/mL noggin (PeproTech), and 100 ng/mL R-spondin (PeproTech). After the organoids were grown in ENR medium with or without Wnt3a for 72 h, they were harvested and total proteins were extracted for western blotting.

### 2.4. RNA Isolation and Real Time Reverse Transcription-PCR (RT-PCR) Analysis

Total RNA isolation and RT-PCR analysis were performed as described previously [32]. Briefly, RT-PCR reactions were performed using cDNA synthesized from 1 µg of total RNA isolated using RNeasy kits (Qiagen, Valencia, CA, USA), High-Capacity cDNA Reverse Transcription Kit (Applied Biosystems, Austin, TX), a TaqMan Gene Expression Master Mix, and TaqMan probes for human *HMGCS2* (assay ID, Hs00985427_m1), *PPAR*γ (assay ID, Hs01115513_m1), *PPAR*α (assay ID, Hs00947539_m1), *FABP2* (assay ID, Hs01573164_g1), *PLIN2* (assay ID, Hs00605340_m1), *IAP* (assay ID, Hs00357579_g1), *SI* (assay ID, Hs00356112_m1), *p21^Waf1^* (assay ID, Hs00355782_m1), *CDX2* (assay ID, Hs01078080_m1), and *GAPDH* (assay ID, Hs99999905_m1) according to instruction provided by the manufacturer (Applied Biosystems).

### 2.5. Chromatin Immunoprecipitation (ChIP) Analysis

Chromatin Immunoprecipitation (ChIP) Assay Kit was obtained from Millipore (Bedford, MA, USA). ChIP assay was performed using an anti-PPARγ antibody or IgG (Santa Cruz Biotechnology) in accordance with the manufacturer’s protocol (Millipore). LS174T cells were transfected with NTC or β-catenin siRNA, and DNA for the LS174T cells was isolated and analyzed by ChIP-qPCR using PerfeCTa SYBR Green SuperMix, ROX (Quantabio, Beverly, MA). The sequence from the *HMGCS2* promoter region containing PPRE was amplified using the primers: forward, 5′-CAGCCATTCCCACACATGCTCA-3′, and reverse, 5′-GACTTTATAAAGCCCCAAGACT-3′. The primers for the distal region of the *IFN-λ1* promoter as non-regulated control: forward, 5′-TTTAAGGGCAGGTGCAGGGTGTC-3′, and reverse, 5′-TTACCCAATGTGGTGGGCACCATC-3′ [34]. ChIP efficiency for an anti-PPARγ antibody or IgG control was shown as a percent of input as described [35,36].

### 2.6. βHB Assay

Intracellular βHB concentration was determined using a Beta-Hydroxybutyrate Assay Kit (MAK041; Sigma-Aldrich) according to the manufacturer’s protocol. Each plotted value was normalized to cell number used from cell lines and total amount of protein used from organoid cultures, respectively.

### 2.7. PPAR*γ* and PPAR*α* Transcription Factor Assays

The DNA-binding activity of PPARγ or PPARα was assessed using PPARγ or PPARα Transcription Factor Assay Kits (Abcam), respectively, according to manufacturer’s instruction. Briefly, the nuclear proteins, extracted using a Nuclear Extraction Kit (Abcam), was added in wells immobilized with specific PPRE sequences. After incubation with the primary anti-PPARs antibody and HRP-conjugated secondary antibody subsequently, the absorbance was measured at 450 nm to determine the transcriptional activity of PPARγ or PPARα.

### 2.8. Measurement of Glycolysis

The Seahorse XF96 Extracellular Flux Analyzer (Agilent, CA, USA) was used by the Redox Metabolism Shared Resource Facility of the University of Kentucky Markey Cancer Center to measure extracellular acidification rate (ECAR) for glycolysis of LS174T cells. The cells transfected with siRNA were seeded at the density of 3 × 10^4^ cells/well in a XF96 plate 24 h before the measurement. The glycolysis stress test was performed according to manufacturer’s protocol and the measurements were normalized to the protein contents in each well. The relative levels of glycolysis and glycolytic capacity, were calculated based on ECAR data obtained in the glycolysis stress tests, using Seahorse Wave software for XF analyzers.

### 2.9. Intestinal Alkaline Phosphatase Activity

Cells were treated with 0, 2.5, 5, and 10 mM 2-DG for the indicated time and intestinal alkaline phosphatase (IAP) activity was determined using Alkaline Phosphatase Yellow (pNPP) Liquid Substrate System (P7998; Sigma-Aldrich) as we have described previously [29].

### 2.10. Statistical Analysis

Bar graphs were generated to represent mean ± SD for each cell culture condition. Relative levels of mRNA, βHB concentration, and transcriptional activity of PPARs and IAP activity were calculated based on mean levels in the NTC group or mean of control cell culture conditions. Fold-changes of western blot densitometry relative to control were calculated for each replicate. Statistical tests were performed using two-sample t-test for relative values and glycolysis ECAR levels, one-sample t-test for western blots, or analysis of variance with contrast statements for pairwise testing or test for linear trend across dose levels. Multiple testing was adjusted using the Holm’s method. *p*-values < 0.05 were considered statistically significant.

## 3. Results

### 3.1. Inhibition of Wnt/β-Catenin Pathway Increased HMGCS2 Expression in Human Intestinal Cancer Cell Lines

**Figure 1:**
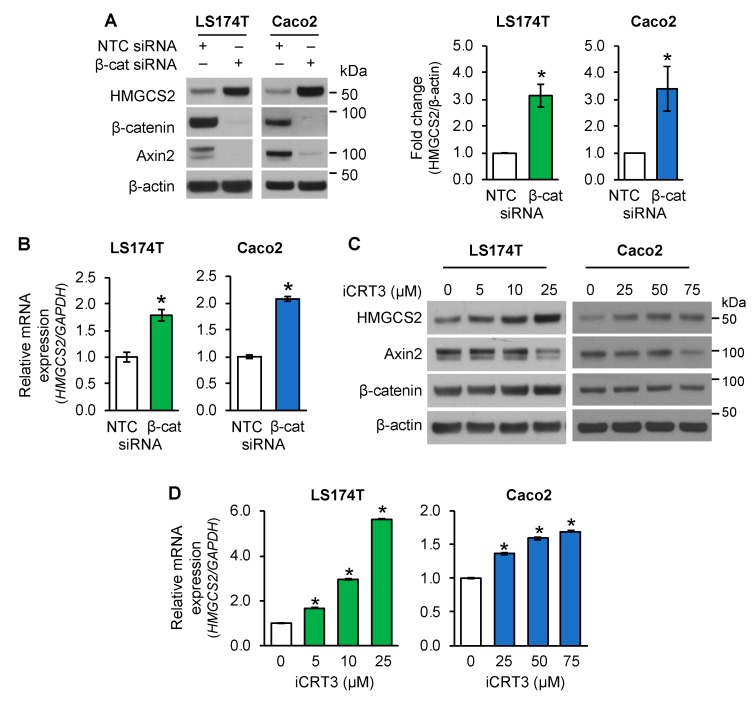
Inhibition of Wnt/β-catenin signaling increased 3-hydroxy-3-methylglutaryl-CoA synthase 2 (HMGCS2) expression in human intestinal cancer cells. (**A**,**B**) LS174T or Caco2 cells transfected with non-target control (NTC) siRNA or β-catenin (β-cat) siRNA were incubated for 48 h. (**A**) Western blot analysis was performed using the antibodies as indicated. HMGCS2 expression from three separate western blots was quantitated densitometrically and is expressed as fold change with respect to β-actin (*n* = 3, data represent mean ± SD; * *p* < 0.05 vs. NTC). (**B**) The level of *HMGCS2* mRNA was assessed by real-time RT-PCR (*n* = 3, data represent mean ± SD; * *p* < 0.05 vs. NTC). (**C**,**D**) Inhibition of Wnt/β-catenin signaling increased the expression of HMGCS2 in LS174T and Caco2 cells. LS174T or Caco2 cells were treated with iCRT3 for 24 h (LS174T) or 48 h (Caco2). (**C**) Western blot analysis was performed using the antibodies as indicated. (**D**) *HMGCS2* mRNA expression was assessed by real time RT-PCR (*n* = 3, data represent mean ± SD; * *p* < 0.05 vs. 0 μM iCRT3).

Wnt/β-catenin signaling plays a critical role in controlling intestinal cell proliferation and differentiation. Recently, we have shown that ketogenesis contributes to intestinal cell differentiation [29]. To determine the regulation of ketogenesis by Wnt/β-catenin signaling in established intestinal cancer cell lines, LS174T and Caco2 cells were transiently transfected with non-targeting control (NTC) siRNA or siRNA targeting β-catenin (β-cat). As shown in Figure 1A, β-catenin knockdown resulted in increased protein expression of the ketogenic enzyme HMGCS2 and decreased expression of Axin2, a well-known target of the Wnt/β-catenin pathway [37], in LS174T and Caco2 cells. Consistently with the increased protein expression, knockdown of β-catenin elevated the expression of *HMGCS2* mRNA in these cells, as detected by real time RT-PCR (Figure 1B).

To further demonstrate the inhibition of the Wnt/β-catenin pathway on HMGCS2 expression, LS174T and Caco2 cells were treated with iCRT3, an inhibitor of Wnt/β-catenin signaling [31,38]. As shown in Figure 1C, treatment with iCRT3 resulted in a dose-dependent induction of HMGCS2 protein expression and decrease of Axin2 protein levels in LS174 and Caco2 cells. In agreement with the increased protein expression, treatment with iCRT3 increased the expression of *HMGCS2* mRNA in these cells, as noted by real time RT-PCR (Figure 1D). Together, these results demonstrate that inhibition of Wnt/β-catenin resulted in increased HMGCS2 expression in LS174 and Caco2 cells.

### 3.2. Activation of Wnt/β-Catenin Signaling Suppressed HMGCS2 Expression in Human Intestinal Cancer Cells and Mouse Small Intestinal Organoids

**Figure 2:**
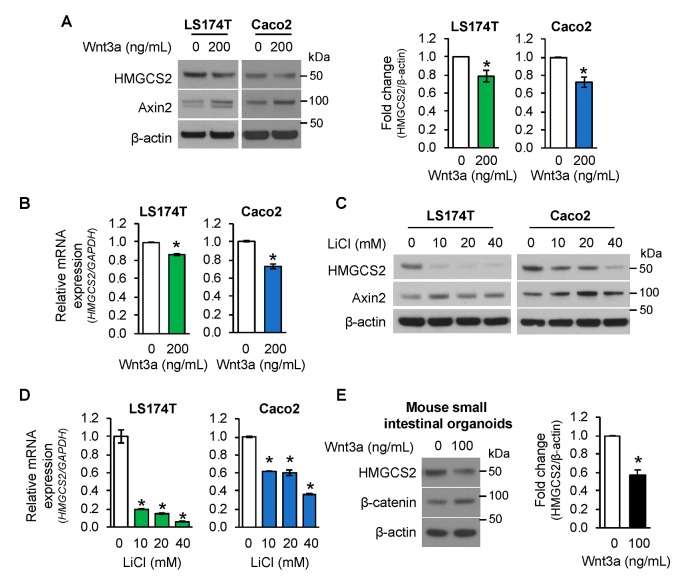
Activation of Wnt/β-catenin pathway suppressed HMGCS2 expression. (**A**,**B**) LS174T and Caco2 cells were treated with 200 ng/mL Wnt3a for 24 h. (**A**) Western blot analysis was performed using the antibodies as indicated. Densitometric quantification from three separate experiments was performed and is represented as fold change with respect to β-actin (*n* = 3, data represent mean ± SD; * *p* < 0.05 vs. 0 ng/mL Wnt3a). (**B**) The level of *HMGCS2* mRNA was determined by real-time RT-PCR. (*n* = 3, data represent mean ± SD; * *p* < 0.05 vs. 0 ng/mL Wnt3a). (**C**,**D**) LS174T and Caco2 cells were treated with various dosages of LiCl or 40 mM of NaCl as control for 24 h. (**C**) Western blot analysis was performed using the antibodies as indicated. (**D**) The level of *HMGCS2* mRNA was determined by real-time RT-PCR. (*n* = 3, data represent mean ± SD; * *p* < 0.05 vs. 40 mM NaCl). (**E**). Mouse small intestinal organoids were treated with 100 ng/mL Wnt3a for 3 days. HMGCS2 protein expression was determined by western blotting. Densitometric analysis from three independent experiments was performed and is represented as fold change with respect to β-actin (*n* = 3, data represent mean ± SD; * *p* < 0.05 vs. 0 ng/mL Wnt3a).

To decipher the regulation of HMGCS2 expression by Wnt activation, LS174T and Caco2 cells were treated with Wnt3a peptide, an activating ligand of the Wnt/β-catenin pathway [31]. As shown in Figure 2A, treatment with Wnt3a activated Wnt/β-catenin signaling as noted by increased expression of Axin2. Importantly, Wnt3a treatment decreased HMGCS2 protein (Figure 2A) and mRNA (Figure 2B) expression in LS174T and Caco2 cells.

To further confirm the decrease of HMGCS2 expression by Wnt/β-catenin pathway activation, LS174 and Caco2 cells were treated with LiCl, which activates Wnt/β-catenin signaling by inhibiting GSK3β activity in normal and colorectal cancer cells [39,40]. In agreement with Wnt3a, treatment with LiCl resulted in a dose-dependent inhibition of HMGCS2 protein (Figure 2C) and mRNA (Figure 2D) expression in these cells. 

To demonstrate the effect of Wnt/β-catenin signaling activation on HMGCS2 expression in normal intestinal cells, mouse small intestinal organoids cultured in Matrigel were incubated with Wnt3a (0 or 100 ng/mL) for 3 days. As shown in Figure 2E, Wnt3a treatment suppressed the expression of HMGCS2 protein, accompanied with an increased level of β-catenin protein. Taken together, our findings demonstrate that Wnt/β-catenin pathway activation suppressed the expression of HMGCS2 in intestinal cells.

### 3.3. Regulation of Ketogenesis by Wnt/β-Catenin Signaling in Intestinal Cancer Cell Lines and Organoids

**Figure 3:**
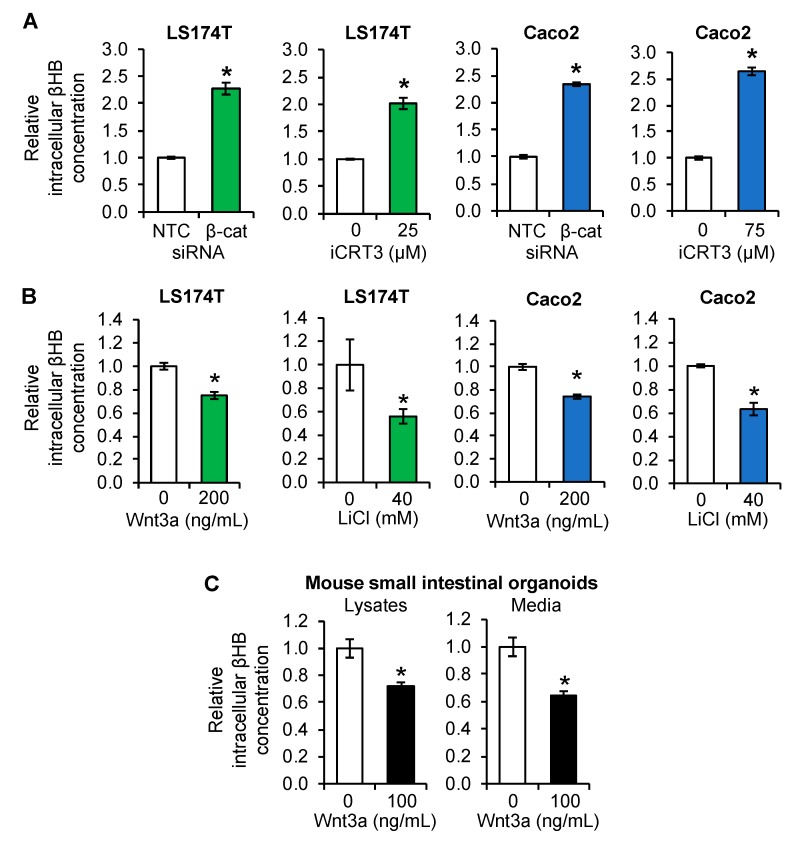
Regulation of β-hydroxybutyrate (βHB) production by Wnt/β-catenin signaling in intestinal cells. (**A**) LS174T and Caco2 cells, transfected with NTC or β-catenin siRNA (β-cat) and incubated for 48 h or treated with iCRT3 for 24 h (LS174T) or 48 h (Caco2), were lysed and βHB content was measured using a βHB assay kit (*n* = 3, data represents mean ± SD; * *p* < 0.05 vs. NTC or 0 μM iCRT3). (**B**) LS174T and Caco2 cells were treated with 200 ng/mL Wnt3a or 40 mM LiCl (40 mM NaCl as control) for 24 h. Cell lysates were used for measurement of βHB content using a βHB assay kit (*n* = 3, data represents mean ± SD; * *p* < 0.05 vs. 0 ng/mL Wnt3a or 40 mM NaCl). (**C**) Mouse small intestinal organoids were treated with 100 ng/mL Wnt3a for 3 days. βHB content in cell lysates and conditioned media was determined using a βHB assay kit (*n* = 3, data represents mean ± SD; * *p* < 0.05 vs. 0 ng/mL Wnt3a).

HMGCS2 plays a pivotal role in the synthesis of ketone bodies [23]. To determine if Wnt/β-catenin-mediated alteration of HMGCS2 expression correlated with alteration of ketone body production, we measured the concentration of βHB, which is the most abundant ketone body [23]. Intracellular βHB content was increased in LS174T and Caco2 cells transfected with β-catenin siRNA or treated with iCRT3 consistently with protein and mRNA expression of HMGCS2 (Figure 3A). In contrast, activation of the Wnt/β-catenin pathway by treatment with Wnt3a or LiCl repressed βHB production in these cells (Figure 3B). Moreover, Wnt3a treatment reduced βHB content in both cell lysates and conditioned media from mouse intestinal organoid cultures (Figure 3C). Together, our findings showed negative regulation of ketogenesis by Wnt/β-catenin signaling in intestinal cells.

### 3.4. Wnt/β-Catenin Signaling Regulated Ketogenesis Independent of c-Myc

**Figure 4:**
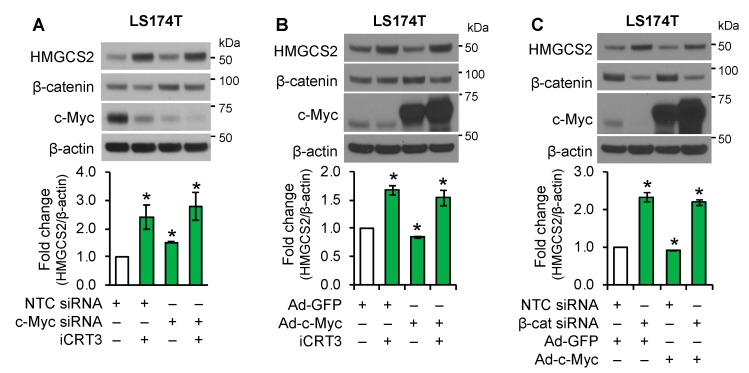
Effect of c-Myc on HMGCS2 regulation by Wnt/β-catenin pathway in LS174T cells. (**A**) LS174T cells transfected with NTC or c-Myc siRNA were treated with iCRT3 for 24 h. (**B**) LS174T cells infected with Ad-GFP control or Ad-c-Myc were treated with iCRT3 (25 μM) for 24 h. (**C**) LS174T cells transfected with NTC or β-catenin were infected with Ad-GFP control or Ad-c-Myc. Western blot analysis was performed using the antibodies as indicated. HMGCS2 expression from three separate western blots was quantitated densitometrically and is expressed as fold change with respect to β-actin (*n* = 3, data represent mean ± SD; * *p* < 0.05 vs. NTC or GFP control).

Previously, it has been reported that HMGCS2 is a target of c-Myc, which suppresses HMGCS2 transcriptional activity [24], suggesting that Wnt/β-catenin signaling may regulate HMGCS2 expression through c-Myc, a known target of the Wnt/β-catenin pathway [8]. To determine whether c-Myc mediates the effect of the Wnt/β-catenin pathway in the regulation of HMGCS2 expression, LS174T cells were transfected with NTC or c-Myc siRNA and then treated with iCRT3. Treatment with iCRT3 dramatically increased HMGCS2 expression and decreased protein expression of c-Myc. However, cells bearing c-Myc siRNA showed more decreased c-Myc expression and only slightly increased basal HMGCS2 expression compared with cells treated with iCRT3 (Figure 4A). These data suggested that iCRT3 did not increase HMGCS2 expression through the repression of c-Myc. To further delineate the role of c-Myc in the regulation of HMGCS2 expression, LS174T cells infected with an adenovirus encoding a GFP control or c-Myc were treated with iCRT3. As shown in Figure 4B, overexpression of c-Myc slightly reduced HMGCS2 expression and, importantly, overexpression of c-Myc did not affect the induction of HMGCS2 by Wnt/β-catenin inhibition, suggesting that c-Myc is not required for Wnt/β-catenin regulation of HMGCS2 expression. Similarly, knockdown of β-catenin increased HMGCS2 expression; this increase was not attenuated by c-Myc overexpression in LS174T cells (Figure 4C). Collectively, our data demonstrated that the regulation of HMGCS2 by the Wnt/β-catenin pathway is not through the regulation of c-Myc expression.

### 3.5. PPAR*γ* Mediated the Role of Wnt/β-Catenin Signaling in the Regulation of Ketogenesis

**Figure 5:**
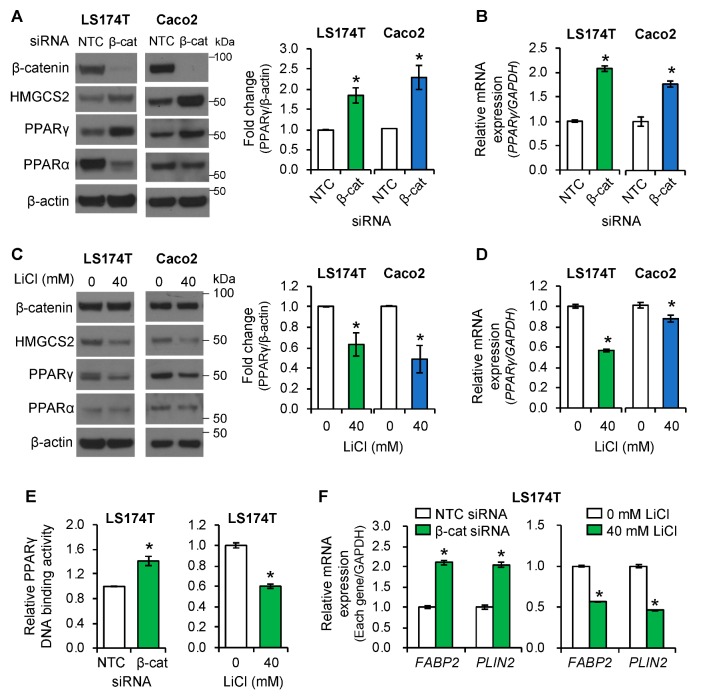
The expression and activity of PPARγ was regulated by the Wnt/β-catenin pathway in intestinal cancer cell lines. (**A**,**B**) LS174T or Caco2 cells were transfected with NTC or β-catenin siRNA and incubated for 48 h. (**A**) Western blot analysis was performed using the antibodies as indicated. PPARγ expression from three independent experiments was quantitated densitometrically and is expressed as fold change with respect to β-actin (*n* = 3, data represent mean ± SD; * *p* < 0.05 vs. NTC). (**B**) *PPAR*γ mRNA was assessed by real-time RT-PCR (*n* = 3, data represent mean ± SD; * *p* < 0.05 vs. NTC). (**C**,**D**) LS174T or Caco2 cells were treated with NaCl (40 mM) as control or LiCl (40 mM) for 24 h. (**C**) Western blotting was performed using the antibodies as indicated. Densitometric analysis from three separate experiments was performed and PPARγ expression is represented as fold change with respect to β-actin (*n* = 3, data represent mean ± SD; * *p* < 0.05 vs. 40 mM/mL NaCl). (**D**) *PPAR*γ mRNA was assessed by real-time RT-PCR (*n* = 3, data represents mean ± SD; * *p* < 0.05 vs. 40 mM/mL NaCl control). (**E**) LS174T cells were transfected with NTC or β-catenin siRNA for 48 h or treated with 40 mM NaCl or 40 mM LiCl for 24 h and PPARγ DNA-binding activity was determined as described under Materials and Methods (*n* = 3, data represents mean ± SD; * *p* < 0.05 vs. NTC or 40 mM NaCl). (**F**) Expression of *FABP2* and *PLIN2* mRNA was assessed by real-time RT-PCR. (*n* = 3, data represents mean ± SD; * *p* < 0.05 vs. NTC or 40 mM/mL NaCl).

PPARα and PPARγ have been shown to control ketogenesis via regulation of HMGCS2 expression [17,18]. To determine whether PPARs play a role in HMGCS2 regulation by Wnt/β-catenin signaling in intestinal cells, we investigated the protein levels of PPARα and γ in LS174T and Caco2 cells transfected with NTC or β-catenin siRNA. As shown in Figure 5A, knockdown of β-catenin increased the protein level of HMGCS2, as expected. Importantly, β-catenin knockdown increased protein (Figure 5A) and mRNA (Figure 5B) levels of PPARγ but not PPARα, in LS174T and Caco2 cells. Conversely, activation of Wnt/β-catenin signaling by treatment with LiCl decreased protein (Figure 5C) and mRNA (Figure 5D) levels of PPARγ in these cells. These results demonstrated the negative regulation of PPARγ expression by Wnt/β-catenin signaling in LS174T and Caco2 cells. We next investigated whether the DNA binding activity of PPARγ and PPARα was regulated by Wnt/β-catenin signaling, using PPARγ or PPARα Transcription Factor Assay Kits. Knockdown of β-catenin increased the DNA binding activity of PPARγ (Figure 5E, left), but not that of PPARα (Figure S1A, left) in LS174T cells. Conversely, activation of the Wnt/β-catenin pathway by treatment with LiCl suppressed PPARγ DNA binding activity (Figure 5E, right) without affecting PPARα DNA binding activity (Figure S1A, right) in LS174T cells. To determine whether increased PPARγ expression and DNA binding activity was correlated with the increased expression of PPARγ target genes in CRC cells, the expressions of *FABP2* (fatty acid binding protein 2) and *PLIN2* (perilipin 2 or adipophilin), two PPARγ target genes [41,42], were evaluated by real time RT-PCR. Knockdown of β-catenin increased, while treatment with LiCl repressed, the expression of *FABP2* and *PLIN2* in LS174T cells (Figure 5F) and the expression of *PLIN2* in Caco2 cells (Figure S1B). Our results demonstrate that inhibition of Wnt/β-catenin signaling increased, while activation of Wnt/β-catenin pathway decreased, the expression and transcriptional activity of PPARγ, but not PPARα, in intestinal cancer cell lines.

**Figure 6:**
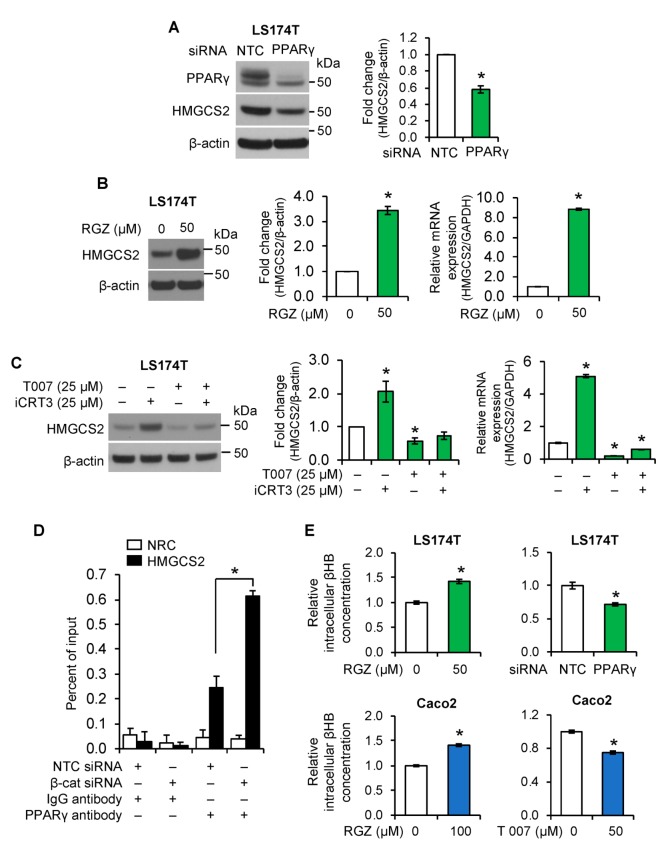
PPARγ regulated HMGCS2 expression in intestinal cancer cell lines. (**A**). LS174T cells were transfected with NTC or PPARγ siRNA. Expression of PPARγ and HMGCS2 was determined by western blot analysis. HMGCS2 expression from three separate western blots was quantitated densitometrically and is expressed as fold change with respect to β-actin (*n* = 3, data represent mean ± SD; * *p* < 0.05 vs. NTC). (**B**,**C**). (**B**) LS174T cells were treated with rosiglitazone (RGZ), an agonist of PPARγ for 24 h; (**C**) LS174T cells were treated with iCRT3 in the presence or absence of T 0070907 (T007), an antagonist of PPARγ for 24 h. Protein expression of HMGCS2 was measured by western blot analysis. Densitometric quantification from three independent experiments was performed and is represented as fold change with respect to β-actin (*n* = 3, data represent mean ± SD; * *p* < 0.05 vs. 40 mM NaCl or vehicle control). The level of *HMGCS2* mRNA was assessed by real-time RT-PCR (*n* = 3, data represents mean ± SD; * *p* < 0.05 vs. control). (**D**). LS174T cells were transfected with NTC or β-catenin siRNA. DNA was extracted and ChIP-qPCR was performed using PPARγ or IgG antibody. The binding efficiency of PPARγ to the promoter regions of *HMGCS2* or *IFN-λ1* (as non-regulated control (NRC)) was analyzed and is represented as a percent of input (*n* = 3, data represents mean ± SD; * *p* < 0.05 vs. NTC). (**E**). LS174T cells were treated with RGZ for 24 h, or transfected with NTC or β-catenin siRNA; Caco2 cells were treated with RGZ or T007 for 24 h. Cells were lysed and βHB content was determined using a βHB assay kit. (*n* = 3, data represents mean ± SD; * *p* < 0.05 vs. control or NTC).

We next determined whether PPARγ regulates HMGCS2 expression in intestinal cells. Knockdown of PPARγ decreased HMGCS expression in LS174T (Figure 6A) and Caco2 cells (Figure S2A). Moreover, treatment with rosiglitazone (RGZ), a highly potent and selective PPARγ agonist [43], significantly increased HMGCS2 protein and mRNA expression in LS174T cells (Figure 6B) and Caco2 cells (Figure S2B, left). Conversely, treatment with T 0070907 (T007), a PPARγ antagonist [44], dramatically decreased basal and attenuated iCRT3-induced HMGCS2 protein and mRNA expression in LS174T (Figure 6C) and Caco2 (Figure S2B, right) cells. These results suggested that PPARγ, downstream of Wnt/β-catenin, mediates the effects of Wnt/β-catenin signaling in regulation of ketogenesis in LS174T and Caco2 cells.

To further demonstrate the transcriptional regulation of *HMGCS2* by Wnt/β-catenin/PPARγ signaling, ChIP-qPCR assay was performed using PPARγ antibody. As shown in Figure 6D, knockdown of β-catenin resulted in increased binding of PPARγ to the *HMGCS2* promoter containing PPAR-binding element (PPRE) in LS174T cells. These findings suggest that Wnt/β-catenin signaling regulates HMGCS2 expression through controlling PPARγ expression and recruitment to the *HMGCS2* promoter.

We have demonstrated the regulation of HMGCS2 expression by Wnt/β-catenin/PPARγ signaling pathway. To determine whether this regulation was correlated with the control of ketone body synthesis, we measured the concentration of βHB. As shown in Figure 6E, activation of PPARγ by treatment with RGZ increased βHB content in LS174T and Caco2 cells. In contrast, inhibition of PPARγ by knockdown of PPARγ or treatment with T007 reduced βHB production in these cells. Taken together, our findings demonstrated that PPARγ mediates the effects of Wnt/β-catenin signaling in regulation of ketogenesis in the intestinal cancer cell lines LS174T and Caco2.

### 3.6. Regulation of Glycolysis by Wnt/β-Catenin/HMGCS2 Pathway

**Figure 7:**
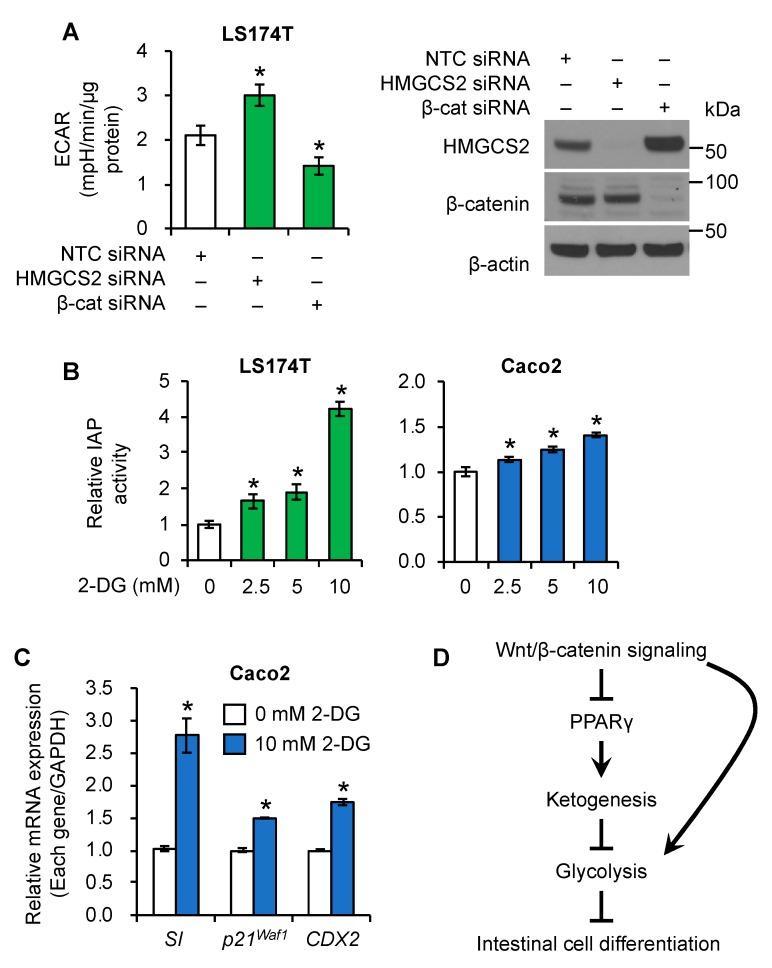
Regulation of glycolysis by Wnt/β-catenin/HMGCS2 pathway. (**A**) LS174T cells transfected with NTC siRNA or siRNA targeting HMGCS2 or β-catenin were subjected to Seahorse Extracellular Flux analysis (left) (*n* = 9, data represent mean ± SD; * *p* < 0.05 vs. NTC). Knockdown of HMGCS2 and β-catenin was confirmed by western blot analysis (right). (**B**,**C**) LS174T cells were treated with 2-DG, a glycolysis inhibitor for 24 h. Caco2 cells were treated with 2-DG for 24 h (RNA) or 48 h (IAP activity assay). (**B**) IAP activity was measured (*n* = 3, data represents mean ± SD; * *p* < 0.05 vs. 0 mM 2-DG). (**C**) Expression of *SI*, *p21^Waf1^* and *CDX2* mRNA was assessed by real-time RT-PCR. (*n* = 3, data represents mean ± SD; * *p* < 0.05 vs. 0 mM 2-DG). (**D**) Inhibition of the Wnt/β-catenin pathway resulted in the increased expression and activation of PPARγ, and thus increased ketogenesis by induction of HMGCS2. Ketogenesis contributes to intestinal cell differentiation via the inhibition of glycolysis.

Emerging evidence indicates that the Wnt/β-catenin pathway controls cellular metabolism, including glycolysis [45,46,47]. In particular, Wnt/β-catenin signaling augments aerobic glycolysis in CRC cells [47]. Results from our laboratory [29] and others [48] have shown that decreased glycolysis is associated with increased intestinal enterocyte differentiation. Moreover, inhibition of glycolysis has been shown to contribute to normal intestinal cell differentiation [33]. To delineate the functional relationship between Wnt/β-catenin signaling and HMGCS2 in regulation of glycolysis, LS174T cells with β-catenin or HMGCS2 knockdown were subjected to Seahorse Extracellular Flux analysis. As shown in Figure 7A and Figure S3A, knockdown of β-catenin reduced glycolysis. Consistently, knockdown of HMGCS2 significantly increased the extracellular acidification rate (ECAR) (Figure 7A), which was associated with increased glycolytic capacity (Figure S3B) in LS174T cells. Intestinal cells can differentiate into an enterocyte-like phenotype characterized by the expression of brush-border enzymes such as intestinal alkaline phosphatase (IAP) and sucrose–isomaltase (SI) [29]. In addition, we found that ketogenesis contributes to intestinal cell differentiation [29]. As we found knockdown of HMGCS2 increased glycolysis, we first determined whether inhibition of glycolysis contributes to differentiation in these CRC cells. Treatment with 2-DG, a glycolysis inhibitor [49] significantly increased IAP activity in LS174T and Caco2 cells (Figure 7B) and induced mRNA levels of SI and IAP in Caco2 and LS174T cells (Figure 7C and Figure S3C), respectively. Moreover, treatment with 2-DG increased mRNA expression of *p21^Waf1^*, which suppresses intestinal cell growth and promotes differentiation [50], and *Caudal-related homeobox transcription factor 2* (*CDX2*), which regulates intestinal epithelium renewal [51], in Caco2 cells (Figure 7C). Taken together, our results suggested a novel role of HMGCS2 in the suppression of glycolysis, which inhibits intestinal cell differentiation.

## 4. Discussion

The Wnt/β-catenin pathway plays a critical role in an extensive range of physiological and pathological processes [8,9,52]. In particular, Wnt/β-catenin signaling is involved in adult epithelial homeostasis, gut development, and pathogenesis of intestinal cells [4,10,11]. Here, we have shown that HMGCS2 is a novel target of the Wnt/β-catenin/PPARγ pathway in intestinal cells. Wnt/β-catenin signaling regulates the expression of HMGCS2 and βHB production in human intestinal cells. Moreover, Wnt/β-catenin signaling modulates ketogenesis through the regulation of PPARγ expression and recruitment to the *HMGCS2* promoter. In addition, knockdown of HMGCS2 increases glycolysis, which regulates differentiation in intestinal cancer cells. Taken together, our results suggest a negative regulation of ketogenesis by Wnt/β-catenin signaling.

Our findings identified HMGCS2 as a novel downstream target gene of Wnt/β-catenin signaling in intestinal cells. A variety of signaling molecules such as Wnt, which is expressed in a gradient along the crypt–villus axis, spatially and temporally regulate a homeostatic balance between proliferation and differentiation in the intestinal epithelium [2,3,4]. Among these factors, EphB receptors and Ephrin-B ligands, which have key roles in regulation of migration and adhesion of cells, are downstream target genes of the Wnt/β-catenin pathway in the intestine [4,53]. In addition, transcription factor *Sox9*, which suppresses differentiation associated genes such as *CDX2* and *Mucin2*, is also regulated by the Wnt/β-catenin pathway [4,54]. Moreover, we found that strong staining of HMGCS2 was detected in small intestinal villi, the most differentiated region of the small bowel mucosa [29]. In contrast, in agreement with the inhibition of HMGCS2 expression by activation of Wnt/β-catenin pathway, activation of Wnt/β-catenin signaling is highest in the bottom part of the crypts and is gradually decreased along the crypt–villus axis [30]. Based on the above studies, our results further suggest a functional relationship between HMGCS2 and Wnt/β-catenin signaling in regulation of intestinal cell proliferation and differentiation.

Recently, we have reported a contributory role of HMGCS2 in intestinal cell differentiation [29]. Increases in ketogenesis by overexpression of HMGCS2 in intestinal cell lines, or in mice fed with a ketone diet, increase intestinal differentiation, while knockdown of HMGCS2 attenuates intestinal cell differentiation [29]. Wnt/β-catenin signaling plays an essential role in maintenance of the intestinal epithelium in the adult organism. Inhibition of Wnt/β-catenin signaling has been shown to block proliferation and increased enterocyte differentiation [55]. In this study, we showed that Wnt/β-catenin signaling negatively regulates the expression of HMGCS2 and βHB production in two intestinal cancer cell lines. These results suggest that the Wnt/β-catenin pathway regulates the balance between proliferation and differentiation, at least in part, through the regulation of ketogenesis. It is likely that altered expression of HMGCS2 is responsible for the impaired equilibrium between intestinal cell proliferation and differentiation associated with several diseases, including CRC, with activation of Wnt/β-catenin signaling. Indeed, our laboratory and others have found that low expressions of HMGCS2 protein were observed in moderately and poorly differentiated colorectal adenocarcinomas compared with well-differentiated tumors [24,56]. Moreover, application of a ketogenic diet delayed CRC cell growth in a mouse xenograft model [57]. Based on previous reports and our current data, HMGCS2, as a target of Wnt/β-catenin signaling, plays a role in maintenance of intestinal cell homeostasis.

We showed that Wnt/β-catenin signaling regulates ketogenesis independently of c-Myc. Oncogenic c-Myc has been shown to inhibit HMGCS2 expression in CRC cells [24]. The c-Myc oncogene, involved in cell proliferation and transformation, is a transcription factor that can alter approximately 15% of genes and can activate or repress the expression of several target genes [58]. Expression of c-Myc is controlled by several growth-promoting factors such as E2F and the Wnt/β-catenin pathway. It is frequently overexpressed in many types of tumors, including CRC [59,60]. Additionally, aberrant activation of Wnt/β-catenin signaling results in the induction of c-Myc expression [61]. Although *HMGCS2* has been identified as a c-Myc target gene, which transcriptionally inhibits *HMGCS2* expression in CRC cells [24], it is unlikely that c-Myc plays a role in the regulation of HMGCS2 expression by Wnt/β-catenin signaling, since either knockdown or overexpression of c-Myc has minor effects on Wnt/β-catenin-regulated HMGCS2 expression. Our results demonstrated that c-Myc is minimally involved in the regulation of HMGCS2 expression by Wnt/β-catenin signaling in intestinal cells.

Our findings showed that the Wnt/β-catenin pathway regulates ketogenesis by targeting PPARγ. PPARs are ligand-activated transcription factors, which regulate important processes in cellular homeostasis such as cell proliferation, differentiation, and metabolism [14,15]. Among the PPAR family, it is well known that PPARα is the major transcription factor responsible for fatty acid metabolism and ketone body biosynthesis via controlling the expression of HMGCS2 [17,23]. With regard to Wnt/β-catenin signaling, PPARγ behaves in an opposite manner and is regulated by the Wnt/β-catenin pathway in colon epithelial cells, and is a key regulator of adipogenesis as well as lipid and glucose metabolism [14,19,20,21]. Moreover, PPARγ is involved in the regulation of cell growth and differentiation in normal and cancer tissues [62,63]. In intestinal epithelial cells, PPARγ inhibits proliferation, increases differentiation, and reduces the size of the proliferative zone for intestinal crypts [64]. High levels of PPARγ correlate with highly differentiated cells at the top of the colonic crypts [65].

Our results demonstrated that Wnt/β-catenin signaling inhibits HMGCS2 expression by suppressing the expression and activation of PPARγ. In agreement with our findings, it has been shown that activity of Wnt/β-catenin pathway presented negative regulation of PPARγ expression and activation in various pathological conditions, including CRCs [20,66,67]. However, it has also been reported that PPARγ-β-catenin interaction seems to increase the stability of the PPARγ protein and enhance PPARγ activity in APC^Min^ mice and in SW480 human colon cancer cells [42]. These controversial intricacies imply that multiple mechanisms may be involved in the regulation of PPARγ expression and its function by Wnt/β-catenin signaling; therefore, further work is needed to clarify this differential effects of Wnt/β-catenin signaling on PPARγ activation.

The results presented here also show that HMGCS2 negatively regulates glycolysis, and inhibition of glycolysis results in increased enterocyte differentiation. The Wnt/β-catenin pathway promotes cell proliferation by altering glycolysis in CRC [45,47]. Inhibition of Wnt/β-catenin signaling decreased, whereas inhibition of ketogenesis increased glycolysis in intestinal cells. Consistently with our results, it has been shown that increased ketogenesis results in decreased glycolytic activity in neurons [68,69]. In addition, results from our laboratory [29] and others [48] have shown that decreased glycolysis is associated with increased intestinal enterocyte differentiation. These results suggest a role for glycolysis in the regulation of intestinal cell differentiation. Indeed, our results showed that inhibition of glycolysis increased intestinal enterocyte differentiation marker expression. Taken together, these results suggest that the Wnt/β-catenin/HMGCS2 axis regulates intestinal cell differentiation through the alteration of glycolysis. 

In summary, we showed that HMGCS2 expression and βHB concentration are regulated by the Wnt/β-catenin/PPARγ pathway in human intestinal cancer cell lines and mouse intestinal organoid cultures. Furthermore, our results suggest that HMGCS2 regulates differentiation through regulation of glycolysis. Our findings identified a functional link between Wnt/β-catenin/PPARγ and ketogenesis in intestinal cells (Figure 7D), suggesting that HMGCS2, acting as a downstream target of the Wnt/β-catenin/PPARγ pathway, contributes to the maintenance of intestinal homeostasis.

## Supplementary Materials

The following are available online at https://www.mdpi.com/2073-4409/8/9/1106/s1, Figure S1: Inhibition or activation of Wnt/β-catenin did not alter PPARα DNA-binding activity. Figure S2: PPARγ regulated HMGCS2 expression in Caco2 cells. Figure S3: HMGCS2 and β-catenin inversely regulated glycolysis in intestinal cancer cells.

## References

[1] Noah T.K., Donahue B., Shroyer N.F. (2011). Intestinal development and differentiation. Exp. Cell Res..

[2] Patterson A.M., Watson A.J.M. (2017). Deciphering the Complex Signaling Systems That Regulate Intestinal Epithelial Cell Death Processes and Shedding. Front. Immunol..

[3] Das D., Fletcher R.B., Ngai J. (2019). Cellular mechanisms of epithelial stem cell self-renewal and differentiation during homeostasis and repair. Wiley Interdiscip. Rev. Dev. Biol..

[4] Scoville D.H., Sato T., He X.C., Li L. (2008). Current view: Intestinal stem cells and signaling. Gastroenterology.

[5] Negroni A., Cucchiara S., Stronati L. (2015). Apoptosis, Necrosis, and Necroptosis in the Gut and Intestinal Homeostasis. Mediat. Inflamm..

[6] Van der Flier L.G., Clevers H. (2009). Stem cells, self-renewal, and differentiation in the intestinal epithelium. Annu. Rev. Physiol..

[7] Van der Heijden M., Vermeulen L. (2019). Stem cells in homeostasis and cancer of the gut. Mol. Cancer.

[8] Klaus A., Birchmeier W. (2008). Wnt signalling and its impact on development and cancer. Nat. Rev. Cancer.

[9] MacDonald B.T., Tamai K., He X. (2009). Wnt/beta-catenin signaling: Components, mechanisms, and diseases. Dev. Cell.

[10] Koch S. (2017). Extrinsic control of Wnt signaling in the intestine. Differentiation.

[11] Krausova M., Korinek V. (2014). Wnt signaling in adult intestinal stem cells and cancer. Cell Signal..

[12] Fearon E.R. (2011). Molecular genetics of colorectal cancer. Annu. Rev. Pathol..

[13] Tan S.H., Barker N. (2018). Wnt Signaling in Adult Epithelial Stem Cells and Cancer. Prog. Mol. Biol. Transl. Sci..

[14] Varga T., Czimmerer Z., Nagy L. (2011). PPARs are a unique set of fatty acid regulated transcription factors controlling both lipid metabolism and inflammation. Biochim. Biophys. Acta.

[15] Fucci A., Colangelo T., Votino C., Pancione M., Sabatino L., Colantuoni V. (2012). The role of peroxisome proliferator-activated receptors in the esophageal, gastric, and colorectal cancer. PPAR Res..

[16] Rodriguez J.C., Gil-Gomez G., Hegardt F.G., Haro D. (1994). Peroxisome proliferator-activated receptor mediates induction of the mitochondrial 3-hydroxy-3-methylglutaryl-CoA synthase gene by fatty acids. J. Biol. Chem..

[17] Grabacka M., Pierzchalska M., Dean M., Reiss K. (2016). Regulation of Ketone Body Metabolism and the Role of PPARalpha. Int. J. Mol. Sci..

[18] Sikder K., Shukla S.K., Patel N., Singh H., Rafiq K. (2018). High Fat Diet Upregulates Fatty Acid Oxidation and Ketogenesis via Intervention of PPAR-gamma. Cell Physiol. Biochem..

[19] Ahmadian M., Suh J.M., Hah N., Liddle C., Atkins A.R., Downes M., Evans R.M. (2013). PPARgamma signaling and metabolism: The good, the bad and the future. Nat. Med..

[20] Lecarpentier Y., Claes V., Vallee A., Hebert J.L. (2017). Thermodynamics in cancers: Opposing interactions between PPAR gamma and the canonical WNT/beta-catenin pathway. Clin. Transl. Med..

[21] Vallee A., Lecarpentier Y., Guillevin R., Vallee J.N. (2018). Opposite Interplay Between the Canonical WNT/beta-Catenin Pathway and PPAR Gamma: A Potential Therapeutic Target in Gliomas. Neurosci. Bull..

[22] Hegardt F.G. (1999). Mitochondrial 3-hydroxy-3-methylglutaryl-CoA synthase: A control enzyme in ketogenesis. Biochem. J..

[23] Newman J.C., Verdin E. (2014). Ketone bodies as signaling metabolites. Trends Endocrinol. Metab..

[24] Camarero N., Mascaro C., Mayordomo C., Vilardell F., Haro D., Marrero P.F. (2006). Ketogenic HMGCS2 is a c-Myc target gene expressed in differentiated cells of human colonic epithelium and down-regulated in colon cancer. Mol. Cancer Res..

[25] Saraon P., Cretu D., Musrap N., Karagiannis G.S., Batruch I., Drabovich A.P., van der Kwast T., Mizokami A., Morrissey C., Jarvi K. (2013). Quantitative proteomics reveals that enzymes of the ketogenic pathway are associated with prostate cancer progression. Mol. Cell Proteom..

[26] Gromov P., Espinoza J.A., Talman M.L., Honma N., Kroman N., Timmermans Wielenga V., Moreira J.M., Gromova I. (2014). FABP7 and HMGCS2 are novel protein markers for apocrine differentiation categorizing apocrine carcinoma of the breast. PLoS ONE.

[27] Tang H., Wu Y., Qin Y., Wang H., Jia Y., Yang S., Luo S., Wang Q. (2017). Predictive significance of HMGCS2 for prognosis in resected Chinese esophageal squamous cell carcinoma patients. Onco Targets Ther..

[28] Su S.G., Yang M., Zhang M.F., Peng Q.Z., Li M.Y., Liu L.P., Bao S.Y. (2017). miR-107-mediated decrease of HMGCS2 indicates poor outcomes and promotes cell migration in hepatocellular carcinoma. Int. J. Biochem. Cell Biol..

[29] Wang Q., Zhou Y., Rychahou P., Fan T.W., Lane A.N., Weiss H.L., Evers B.M. (2017). Ketogenesis contributes to intestinal cell differentiation. Cell Death Differ..

[30] Spit M., Koo B.K., Maurice M.M. (2018). Tales from the crypt: Intestinal niche signals in tissue renewal, plasticity and cancer. Open Biol..

[31] Kim J.T., Liu C., Zaytseva Y.Y., Weiss H.L., Townsend C.M., Evers B.M. (2015). Neurotensin, a novel target of Wnt/beta-catenin pathway, promotes growth of neuroendocrine tumor cells. Int. J. Cancer.

[32] Kim J.T., Li J., Song J., Lee E.Y., Weiss H.L., Townsend C.M., Evers B.M. (2015). Differential expression and tumorigenic function of neurotensin receptor 1 in neuroendocrine tumor cells. Oncotarget.

[33] Rodriguez-Colman M.J., Schewe M., Meerlo M., Stigter E., Gerrits J., Pras-Raves M., Sacchetti A., Hornsveld M., Oost K.C., Snippert H.J. (2017). Interplay between metabolic identities in the intestinal crypt supports stem cell function. Nature.

[34] Thomson S.J.P., Goh F.G., Banks H., Krausgruber T., Kotenko S.V., Foxwell B.M.J., Udalova I.A. (2009). The role of transposable elements in the regulation of IFN-lambda1 gene expression. Proc. Natl. Acad. Sci. USA.

[35] Lacazette E. (2017). A laboratory practical illustrating the use of the ChIP-qPCR method in a robust model: Estrogen receptor alpha immunoprecipitation using Mcf-7 culture cells. Biochem. Mol. Biol. Educ.

[36] Lin X., Tirichine L., Bowler C. (2012). Protocol: Chromatin immunoprecipitation (ChIP) methodology to investigate histone modifications in two model diatom species. Plant Methods.

[37] Jho E.H., Zhang T., Domon C., Joo C.K., Freund J.N., Costantini F. (2002). Wnt/beta-catenin/Tcf signaling induces the transcription of Axin2, a negative regulator of the signaling pathway. Mol. Cell Biol..

[38] Gonsalves F.C., Klein K., Carson B.B., Katz S., Ekas L.A., Evans S., Nagourney R., Cardozo T., Brown A.M., DasGupta R. (2011). An RNAi-based chemical genetic screen identifies three small-molecule inhibitors of the Wnt/wingless signaling pathway. Proc. Natl. Acad. Sci. USA.

[39] Caspi M., Zilberberg A., Eldar-Finkelman H., Rosin-Arbesfeld R. (2008). Nuclear GSK-3beta inhibits the canonical Wnt signalling pathway in a beta-catenin phosphorylation-independent manner. Oncogene.

[40] Vidal F., de Araujo W.M., Cruz A.L., Tanaka M.N., Viola J.P., Morgado-Diaz J.A. (2011). Lithium reduces tumorigenic potential in response to EGF signaling in human colorectal cancer cells. Int. J. Oncol..

[41] Gupta R.A., Brockman J.A., Sarraf P., Willson T.M., DuBois R.N. (2001). Target genes of peroxisome proliferator-activated receptor gamma in colorectal cancer cells. J. Biol. Chem..

[42] Jansson E.A., Are A., Greicius G., Kuo I.C., Kelly D., Arulampalam V., Pettersson S. (2005). The Wnt/beta-catenin signaling pathway targets PPARgamma activity in colon cancer cells. Proc. Natl. Acad. Sci. USA.

[43] Dang Y.F., Jiang X.N., Gong F.L., Guo X.L. (2018). New insights into molecular mechanisms of rosiglitazone in monotherapy or combination therapy against cancers. Chem. Biol. Interact..

[44] Burton J.D., Goldenberg D.M., Blumenthal R.D. (2008). Potential of peroxisome proliferator-activated receptor gamma antagonist compounds as therapeutic agents for a wide range of cancer types. PPAR Res..

[45] Pate K.T., Stringari C., Sprowl-Tanio S., Wang K., TeSlaa T., Hoverter N.P., McQuade M.M., Garner C., Digman M.A., Teitell M.A. (2014). Wnt signaling directs a metabolic program of glycolysis and angiogenesis in colon cancer. EMBO J..

[46] Thompson C.B. (2014). Wnt meets Warburg: Another piece in the puzzle?. EMBO J..

[47] Sherwood V. (2015). WNT signaling: An emerging mediator of cancer cell metabolism?. Mol. Cell Biol..

[48] Stringari C., Edwards R.A., Pate K.T., Waterman M.L., Donovan P.J., Gratton E. (2012). Metabolic trajectory of cellular differentiation in small intestine by Phasor Fluorescence Lifetime Microscopy of NADH. Sci. Rep..

[49] Zhang D., Li J., Wang F., Hu J., Wang S., Sun Y. (2014). 2-Deoxy-D-glucose targeting of glucose metabolism in cancer cells as a potential therapy. Cancer Lett..

[50] Evers B.M. (1999). Intestinal cell differentiation: Cellular mechanisms and the search for the perfect model focus on “involvement of p21(WAF1/Cip1) and p27(Kip1) in intestinal epithelial cell differentiation”. Am. J. Physiol..

[51] Saad R.S., Ghorab Z., Khalifa M.A., Xu M. (2011). CDX2 as a marker for intestinal differentiation: Its utility and limitations. World J. Gastrointest. Surg..

[52] Kim J.T., Li J., Jang E.R., Gulhati P., Rychahou P.G., Napier D.L., Wang C., Weiss H.L., Lee E.Y., Anthony L. (2013). Deregulation of Wnt/beta-catenin signaling through genetic or epigenetic alterations in human neuroendocrine tumors. Carcinogenesis.

[53] Batlle E., Henderson J.T., Beghtel H., van den Born M.M., Sancho E., Huls G., Meeldijk J., Robertson J., van de Wetering M., Pawson T. (2002). Beta-catenin and TCF mediate cell positioning in the intestinal epithelium by controlling the expression of EphB/ephrinB. Cell.

[54] Blache P., van de Wetering M., Duluc I., Domon C., Berta P., Freund J.N., Clevers H., Jay P. (2004). SOX9 is an intestine crypt transcription factor, is regulated by the Wnt pathway, and represses the CDX2 and MUC2 genes. J. Cell Biol..

[55] Fevr T., Robine S., Louvard D., Huelsken J. (2007). Wnt/beta-catenin is essential for intestinal homeostasis and maintenance of intestinal stem cells. Mol. Cell Biol..

[56] Wang Q., Zhou Y., Rychahou P., Harris J.W., Zaytseva Y.Y., Liu J., Wang C., Weiss H.L., Liu C., Lee E.Y. (2018). Deptor is a novel target of Wnt/beta-catenin/c-Myc and contributes to colorectal cancer cell growth. Cancer Res..

[57] Hao G.W., Chen Y.S., He D.M., Wang H.Y., Wu G.H., Zhang B. (2015). Growth of human colon cancer cells in nude mice is delayed by ketogenic diet with or without omega-3 fatty acids and medium-chain triglycerides. Asian Pac. J. Cancer Prev..

[58] Dang C.V., O’Donnell K.A., Zeller K.I., Nguyen T., Osthus R.C., Li F. (2006). The c-Myc target gene network. Semin. Cancer Biol..

[59] Sipos F., Firneisz G., Muzes G. (2016). Therapeutic aspects of c-MYC signaling in inflammatory and cancerous colonic diseases. World J. Gastroenterol..

[60] Poole C.J., van Riggelen J. (2017). MYC-Master Regulator of the Cancer Epigenome and Transcriptome. Genes.

[61] Rennoll S., Yochum G. (2015). Regulation of MYC gene expression by aberrant Wnt/beta-catenin signaling in colorectal cancer. World J. Biol. Chem..

[62] Sabatino L., Pancione M., Votino C., Colangelo T., Lupo A., Novellino E., Lavecchia A., Colantuoni V. (2014). Emerging role of the beta-catenin-PPARgamma axis in the pathogenesis of colorectal cancer. World J. Gastroenterol..

[63] Michalik L., Desvergne B., Wahli W. (2004). Peroxisome-proliferator-activated receptors and cancers: Complex stories. Nat. Rev. Cancer.

[64] Bajwa P.J., Lee J.W., Straus D.S., Lytle C. (2009). Activation of PPARgamma by rosiglitazone attenuates intestinal Cl- secretion. Am. J. Physiol. Gastrointest. Liver Physiol..

[65] Mansen A., Guardiola-Diaz H., Rafter J., Branting C., Gustafsson J.A. (1996). Expression of the peroxisome proliferator-activated receptor (PPAR) in the mouse colonic mucosa. Biochem. Biophys. Res. Commun..

[66] Lecarpentier Y., Claes V., Vallee A., Hebert J.L. (2017). Interactions between PPAR Gamma and the Canonical Wnt/Beta-Catenin Pathway in Type 2 Diabetes and Colon Cancer. PPAR Res..

[67] Vallee A., Lecarpentier Y. (2018). Crosstalk Between Peroxisome Proliferator-Activated Receptor Gamma and the Canonical WNT/beta-Catenin Pathway in Chronic Inflammation and Oxidative Stress During Carcinogenesis. Front. Immunol..

[68] Lund T.M., Ploug K.B., Iversen A., Jensen A.A., Jansen-Olesen I. (2015). The metabolic impact of beta-hydroxybutyrate on neurotransmission: Reduced glycolysis mediates changes in calcium responses and KATP channel receptor sensitivity. J. Neurochem..

[69] Yellen G. (2008). Ketone bodies, glycolysis, and KATP channels in the mechanism of the ketogenic diet. Epilepsia.

