# Supplementary material for: Regulation of Ketogenic Enzyme HMGCS2 by Wnt/β-catenin/PPARγ Pathway in Intestinal Cells
Source: Cells. 2019 Sep 19;8(9):1106. doi: 10.3390/cells8091106 (PMC6770209; doi:10.3390/cells8091106)
Supplement: Supplementary file 1 [file cells-08-01106-s001.pdf]

## Supplementary Material

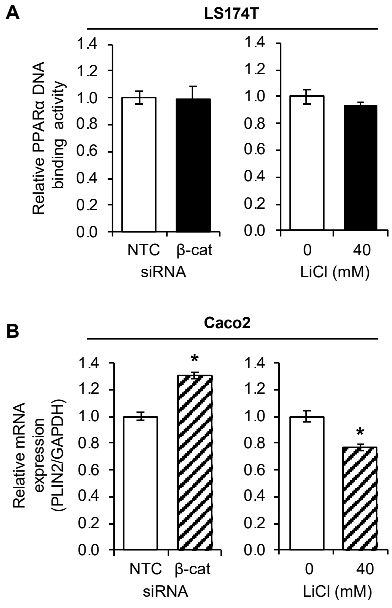


**Figure S1.** Inhibition or activation of Wnt/β-catenin did not alter PPAR$\alpha$ DNA-binding activity. **(A)** LS174T cells were transfected with NTC or β-catenin siRNA (left) for 48 h or treated with 40 mg/ml NaCl as control or 40 mM LiCl (right) for 24 h. PPAR$\alpha$ DNA-binding activity was determined through PPAR$\alpha$ ELISA assays as described under Materials and Methods (n=3, data represents mean ± SD). **(B)** Expression of PLIN2 mRNA was assessed by real-time RT-PCR (n=3, data represents mean ± SD; *$p < 0.05$ vs NTC or 40 mg/ml NaCl).

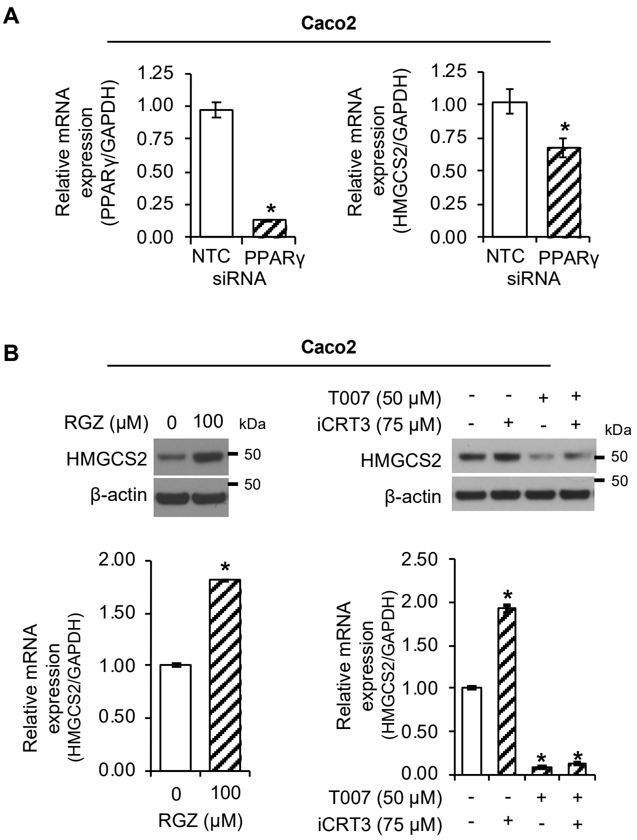


**Figure S2. PPARγ regulated HMGCS2 expression in Caco2 cells. (A)** Caco2 cells were transfected with NTC or PPARγ siRNA. Expression of PPARγ (left) and HMGCS2 (right) mRNA was determined by real-time RT-PCR (n=3, data represents mean ± SD; $^*p < 0.05$ vs NTC). **(B)** Caco2 cells were treated with RGZ for 24 h (left); Caco2 cells were treated iCRT3 for 24 h followed by combination with or without T 0070907 (T007) for additional 24 h (right). Protein level of HMGCS2 was measured by Western blot analysis (upper). The level of HMGCS2 mRNA was assessed by real-time RT-PCR (lower) (n=3, data represents mean ± SD; $^*p < 0.05$ vs control).

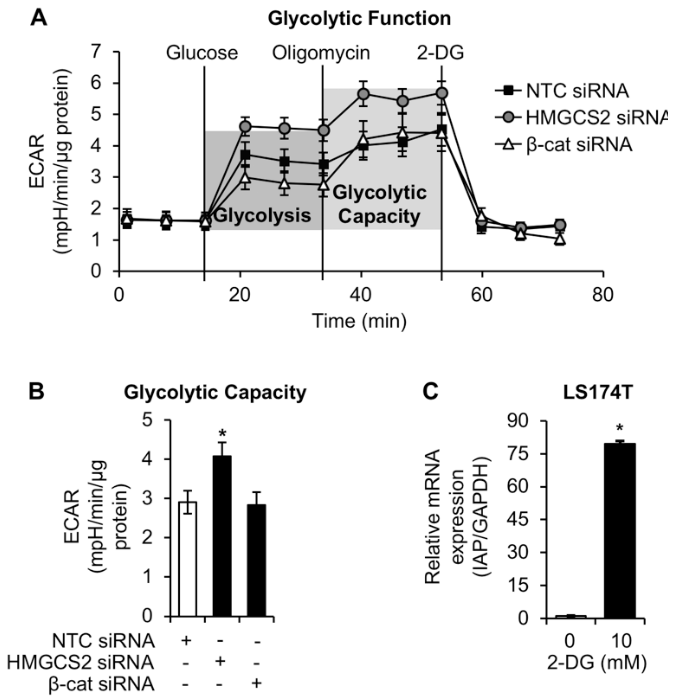


**Figure S3. HMGCS2 and β-catenin inversely regulated glycolysis in intestinal cells. (A)** Representative ECAR measurements obtained from the glycolysis stress test performed in LS174T cells transfected with HMGCS2 or β-catenin siRNA using the Seahorse XF96 Extracellular Flux analyzer (Agilent). Glucose, oligomycin and 2-DG were added at the indicated points. **(B)** ECAR associated with glycolytic capacity was calculated based on the measurements obtained upon the addition of individual compounds (n=9, data represents mean ± SD; *p < 0.05 vs NTC). **(C)** LS174T cells were treated with 2-DG for 24 h. Expression of IAP mRNA was assessed by real-time RT-PCR (n=3, data represents mean ± SD; *p < 0.05 vs 0 mM 2-DG).
